# Trunnionosis in Metal-on-Polyethylene Total Hip Arthroplasty With Periprosthetic Infection: A Case Report

**DOI:** 10.7759/cureus.49401

**Published:** 2023-11-25

**Authors:** Fumito Kobayashi, Kenichi Oe, Shohei Sogawa, Tomohisa Nakamura, Takanori Saito

**Affiliations:** 1 Department of Orthopaedic Surgery, Kansai Medical University, Hirakata, JPN

**Keywords:** trunnionosis, total hip arthroplasty (tha), periprosthetic joint infection, metal-on-polyethylene, adverse reaction to metal debris

## Abstract

A 67-year-old man who underwent right hemiarthroplasty and left total hip arthroplasty (THA) experienced left hip pain two years previously. No previous diagnosis was made at other hospitals. Radiography revealed left hip trunnionosis because of stem-neck shortening, with periprosthetic joint infection (PJI) spreading to both hips. Bilateral revision THA was performed, but the treatment was difficult due to the delayed diagnosis, necessitating the extraction of the well-fixed stem for PJI. Trunnionosis is caused by implant-related, surgical, and patient factors, and early diagnosis is important because of its association with PJI. Furthermore, even implants with few reports of trunnionosis can lead to this complication. Surgeons should always consider that performing THA using a large-diameter head predisposes the patient to trunnionosis.

## Introduction

Since 1990, metal-on-metal (MoM) total hip arthroplasty (THA) has been widely used to develop resurfacing and large-diameter heads. It was reported that MoM caused complications, such as postoperative pain, osteonecrosis, and soft tissue necrosis due to metal wear particles and metal ions around 2006. Abnormal tissue reactions around the THA are called adverse reactions to metal debris (ARMD), adverse local tissue reactions, aseptic lymphocyte-dominated vasculitis-associated lesions, and pseudotumors. However, corrosion at the head-neck junction, first reported in 1991 and later termed “trunnionosis”, could also result in tissue damage [1-4]. Therefore, ARMD can occur in MoM THAs and on the surface of other materials, and trunnionosis has been reported in metal-on-polyethylene (MoP) THAs [2-6].

We report a rare case of trunnionosis after MoP THAs, with bilateral periprosthetic joint infection (PJI). The implants we used were not reported in the review of the Japanese Society for Replacement Arthroplasty [7]. Delayed trunnionosis diagnosis was a problem in the case; thus, we believe the trunnionosis concept should be increasingly popularized to avoid it being overlooked.

## Case presentation

A 67-year-old man underwent left THA (Cup: Trident X3 polyethylene liner; Head: cobalt-chromium-molybdenum alloy (CoCr) 40 mm; Stem: Accolade TMZF, Stryker, Mahwah, USA) 13 years previously and right hemiarthroplasty (Stem: Accolade 2, Stryker) 6 years previously for osteonecrosis of the bilateral femoral heads at hospital A. Two years previously, he experienced left hip pain and visited hospital B, where he was diagnosed with heterotopic ossification via radiography. As the pain did not reduce, he visited the Department of General Medicine at hospital B and was diagnosed with “atypical osteomyelitis” after a thorough examination, including needle biopsy and positron emission tomography. A year later, he visited hospital C because of pus drainage and underwent wound debridement on the left hip. He consulted us because intraoperative findings revealed marked metallosis and debris. The patient had no other medical history.

**Figure 1:**
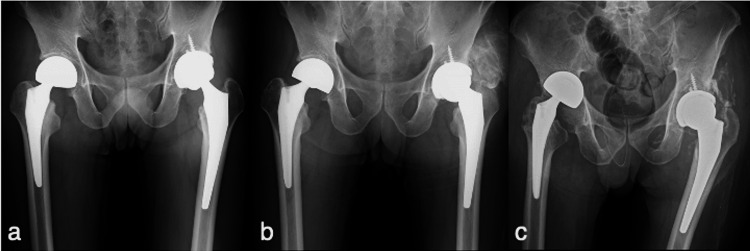
Anteroposterior radiograph. (a) At seven years after left total hip arthroplasty (THA) and three months after right hemiarthroplasty. (b) At 11 years after THA and four years after hemiarthroplasty. (c) At 13 years after THA and six years after hemiarthroplasty.

On admission, the patient had difficulty walking because of pain. He could not stand without support, and his muscle strength had clearly deteriorated. The wound was mildly erythematous. Radiography revealed no abnormalities until 7 years postoperatively (Figure 1a). Eleven years after the first surgery, stem-neck shortening of the left THA and heterotopic ossification were observed around the gluteal muscles (Figure 1b). We observed right hemiarthroplasty migration when he presented to us 13 years after the first surgery (Figure 1c).

**Figure 2:**
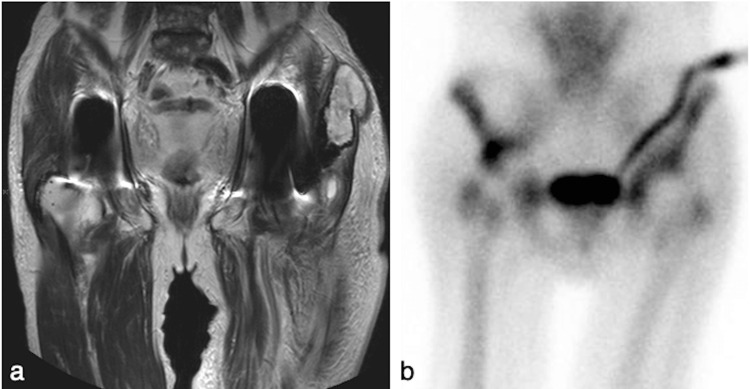
Preoperative imaging. (a) T2-weighted magnetic resonance imaging showed a pseudotumor in the left hip. (b) Bone scintigraphy showed uptake in both hips.

Computed tomography (CT) showed metal deposits around the left hip joint without fluid retention. Magnetic resonance imaging (MRI) at hospital B revealed a pseudotumor with low T1 and high T2 signals in the gluteus medius muscle (Figure 2a). Bone scintigraphy showed uptake in both hip joints (Figure 2b). Preoperative laboratory examination showed a white blood cell (WBC) count of 12,200/mm^3^ and a C-reactive protein (CRP) level of 11.97 mg/dL. *Staphylococcus aureus* and *Pseudomonas aeruginosa* were identified in joint fluid cultures at hospital C. Since there was also right hemiarthroplasty migration in the patient, bilateral PJI was diagnosed, with suspected left hip trunnionosis.

**Figure 3:**
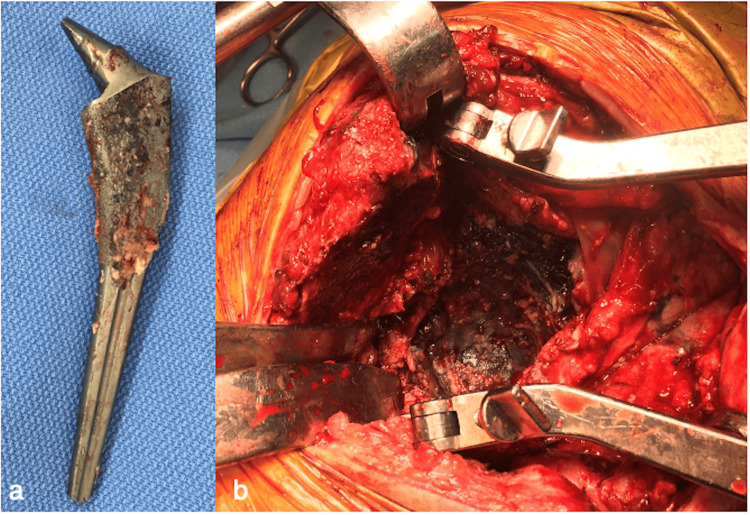
Intraoperative photographs. (a) Stem appearance showed the deformity of the trunnion. (b) Metal debris was observed between the cup and the acetabulum.

The patient underwent emergency bilateral revision THAs. Intraoperative findings showed no fluid retention or tissue contamination in the right hip. One-stage revision THA (KT plate; K-MAX CLHO flanged cup; 26-mm metal head; SC stem; Palacos G bone cement, KYOCERA Medical, Osaka, Japan) was performed. Migration due to low-grade PJI was diagnosed, although joint fluid culture was not performed preoperatively. No obvious pseudotumor or pus exudation was noted in the left hip owing to the recent debridement. However, much metallic debris adhered to the gluteus medius and other soft tissues. No obvious muscle atrophy was observed. Since the stem neck was deformed and the head could be easily extracted, trunnionosis was diagnosed (Figure 3a). The stem fixation was good. After cup extraction on the acetabular side, much metal debris was observed between the cup and the acetabulum (Figure 3b). The hip joint was exhaustively flushed and irrigated with a hydrogen peroxide solution and povidone-iodine.

**Figure 4:**
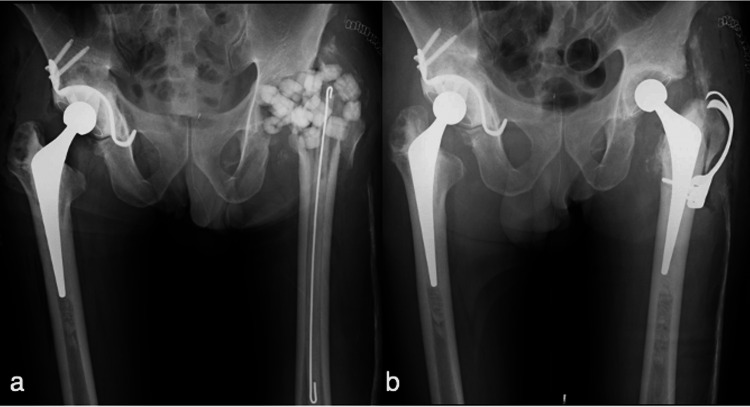
Anteroposterior radiograph. (a) Right-hip one-stage revision total hip arthroplasty (THA) was performed, and antibiotic-loaded acrylic cement beads and rod were deposited in the left hip after bilateral debridement. (b) Left-hip two-stage revision THA was performed 6.5 weeks later.

Subsequently, antibiotic-loaded acrylic cement (ALAC) beads and rod, including 0.5 g gentamicin sulfate, 2.0 g vancomycin hydrochloride, and 1.0 g meropenem hydrate (MEPM) per 40 g cement, were deposited (Figure 4a), followed immediately by 12-hourly and 24-hourly 0.5 g MEPM and 0.35 g daptomycin intravenous injection, respectively. Intraoperative culture results revealed *Staphylococcus aureus* in both hip joints with bacterial susceptibility to methicillin, which was subsequently changed to levofloxacin hydrate. Only the right hip was allowed full weight-bearing movement on the second postoperative day. At 6.5 weeks after the first thorough debridement, clinical signs and CRP levels were reviewed, and a second thorough debridement was performed before THA. Two-stage revision THA (K-MAX CLHO flanged cup; 26-mm metal head; SC stem; Palacos G bone cement, KYOCERA Medical; Trochanteric hook plate, Teijin Nakashima Medical, Okayama, Japan)was performed in the left hip using ALAC comprising 0.5 g gentamicin sulfate and 1.5 g vancomycin hydrochloride per 40 g cement (Figure 4b).

**Figure 5:**
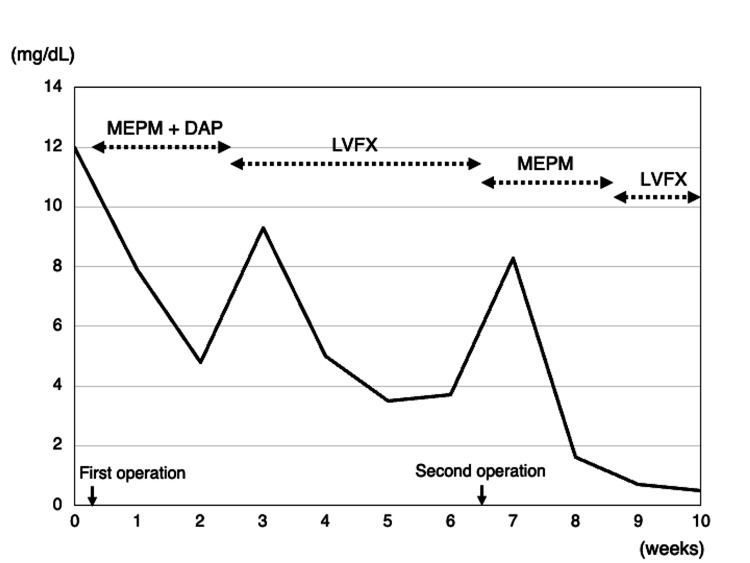
Progression of C-reactive protein level and treatment course. CRP: C-reactive protein, DPT: daptomycin, LVFX: levofloxacin, MEPM: meropenem trihydrate.

This was followed immediately by a 12-hourly 0.5 g MEPM intravenous injection. Full weight bearing was allowed for the left hip on the second postoperative day, and oral antibiotic therapy was administered for 3 months (Figure 5). At 1 year after the revision THAs, no recurrent symptoms were noted in both hips.

## Discussion

Trunnionosis is primarily caused by implant-related, surgical, and patient factors. Implant-related factors include using implants with a large head or taper design and structure, bending stiffness, material and surface finish, and using mismatched metals between the head and stem [8]. These factors result in a mechanically assisted crevice corrosion phenomenon, leading to pain, swelling, joint instability, and cytotoxicity [5]. In the present case, the head was made of cobalt and chromium and it had a large diameter (approx. 40 mm), and the stem was made of Ti-12Mo-6Zr-2Fe (TMZF); thus, the head and stem were made of different metals. Furthermore, according to an experimental study of the relationship between taper geometry and metal stiffness in trunnions, the V40 geometry and TMZF combination had the worst stiffness [8]. Therefore, it can be inferred that the implant makeup was predisposed to trunnionosis, although this condition can be caused by multiple factors.

ARMD diagnosis is made by confirming that the patient is experiencing intense pain in the groin, buttock, thigh, and around the adductor area, and that blood draws show elevated serum cobalt and chromium levels, erythrocyte sedimentation rate 32.0 mm/h, and CRP 10.0 mg/L or higher, without infection. Since radiography does not indicate the initial changes, soft tissue damage should be evaluated by ultrasound, CT, and MRI. However, trunnionosis of MoP should be assessed with caution because the symptoms are less severe than those of MoM [5]. Furthermore, it is important to carefully evaluate radiography images in asymptomatic patients before a trunnionosis diagnosis is made. Serum cobalt and chromium should be measured if trunnionosis is suspected, and the tests are covered by health insurance, although they are not routine tests. If trunnionosis is diagnosed, revision surgery should be performed even for asymptomatic patients because ARMD can be generated in these cases, complicating treatment. Regarding MoM treatment, Japanese guidelines advocate revision surgery if radiography shows implant loosening. Even in the absence of loosening, frequent follow-up is recommended if there are abnormal MRI findings, and revision surgery is required if there are no abnormal radiography findings but there are clinical symptoms and abnormal MRI findings. Annual follow-up is recommended for patients without abnormal MRI findings [9]. According to a report by the Ministry of Health, Labor, and Welfare in Japan, ARMD occurred with implants such as Conserve (Wright Medical Technology, Arlington, USA; 9.0%), Cormet (Corin, Cirencester, Gloucestershire, UK; 8.5%), and Adept (Stryker; 4.0%), Ultamet (DePuy International, Leeds, UK; 3.1%) [9], but the list does not include Accolade. However, the Australian government alerted the manufacturing company of Accolade implants in 2016, and there have been case reports [6,10-14]. The actual incidence of ARMDs may be much higher than that in the literature because incriminating information regarding surgeons or manufacturers may have been covered. Accolade was later remodeled into Accolade 2, and the design and material were changed to Ti-6Al-4V. Notably, even implants with few reports of their association with ARMD can cause ARMD.

PJI has been commonly associated with ARMD, suggesting an association between metallosis and PJI [15-18]. Furthermore, CoCr particles reportedly promoted biofilm formation, which could cause infection [19]. In the present case, soft tissue evaluation was performed with MRI after pain onset, and a diagnosis was not made despite the presence of a pseudotumor. The resulting PJI led to the referral and ARMD diagnosis, but a two-stage revision THA had to be performed. The delayed trunnionosis diagnosis might have caused the PJI, which then spread to the contralateral side. The Regulatory Agency in the UK in 2010 and the Food and Drug Administration in the US in 2012 issued alerts, including follow-up recommendations for ARMD, and an algorithm was proposed in Japan in 2016. However, based on our experience of this case, even orthopedic surgeons are unaware of trunnionosis. Since hemiarthroplasty is performed in many hospitals and trunnionosis is a complication of hemiarthroplasty, information on this clinical possibility should be thoroughly disseminated. Poor outcomes have also been reported for revision THA performed after ARMD progression [20]. Given the high likelihood of PJI developing, revision THA should be strongly considered upon ARMD diagnosis.

## Conclusions

Early diagnosis of trunnionosis is important because trunnionosis is frequently associated with PJI. Even implants with few reports of trunnionosis can lead to this complication. Therefore, surgeons should always consider that performing THA with an implant with a large head can lead to trunnionosis.
